# Optimization of DNA extraction for advancing coral microbiota investigations

**DOI:** 10.1186/s40168-017-0229-y

**Published:** 2017-02-08

**Authors:** Laura Weber, Emelia DeForce, Amy Apprill

**Affiliations:** 1 Massachusetts Institute of Technology-Woods Hole Oceanographic Institution Joint Program in Oceanography/Applied Ocean Science and Engineering, Cambridge, MA 02139 USA; 2 Woods Hole Oceanographic Institution, Marine Chemistry and Geochemistry, Woods Hole, MA 02543 USA; 3 MO BIO Laboratories, Inc., Carlsbad, CA 92010 USA

**Keywords:** Coral microbiota, DNA extraction, Optimization, SSU ribosomal RNA gene, Amplicon sequencing

## Abstract

**Background:**

DNA-based sequencing approaches are commonly used to identify microorganisms and their genes and document trends in microbial community diversity in environmental samples. However, extraction of microbial DNA from complex environmental samples like corals can be technically challenging, and extraction methods may impart biases on microbial community structure.

**Methods:**

We designed a two-phase study in order to propose a comprehensive and efficient method for DNA extraction from microbial cells present in corals and investigate if extraction method influences microbial community composition. During phase I, total DNA was extracted from seven coral species in a replicated experimental design using four different MO BIO Laboratories, Inc., DNA Isolation kits: PowerSoil®, PowerPlant® Pro, PowerBiofilm®, and UltraClean® Tissue & Cells (with three homogenization permutations). Technical performance of the treatments was evaluated using DNA yield and amplification efficiency of small subunit ribosomal RNA (SSU ribosomal RNA (rRNA)) genes. During phase II, potential extraction biases were examined via microbial community analysis of SSU rRNA gene sequences amplified from the most successful DNA extraction treatments.

**Results:**

In phase I of the study, the PowerSoil® and PowerPlant® Pro extracts contained low DNA concentrations, amplified poorly, and were not investigated further. Extracts from PowerBiofilm® and UltraClean® Tissue and Cells permutations were further investigated in phase II, and analysis of sequences demonstrated that overall microbial community composition was dictated by coral species and not extraction treatment. Finer pairwise comparisons of sequences obtained from *Orbicella faveolata*, *Orbicella annularis*, and *Acropora humilis* corals revealed subtle differences in community composition between the treatments; PowerBiofilm®-associated sequences generally had higher microbial richness and the highest coverage of dominant microbial groups in comparison to the UltraClean® Tissue and Cells treatments, a result likely arising from using a combination of different beads during homogenization.

**Conclusions:**

Both the PowerBiofilm® and UltraClean® Tissue and Cells treatments are appropriate for large-scale analyses of coral microbiota. However, studies interested in detecting cryptic microbial members may benefit from using the PowerBiofilm® DNA treatment because of the likely enhanced lysis efficiency of microbial cells attributed to using a variety of beads during homogenization. Consideration of the methodology involved with microbial DNA extraction is particularly important for studies investigating complex host-associated microbiota.

**Electronic supplementary material:**

The online version of this article (doi:10.1186/s40168-017-0229-y) contains supplementary material, which is available to authorized users.

## Background

The coral holobiont [1, 2] consists of a network of interacting bacterial, archaeal, viral, fungal, protistan (i.e., *Symbiodinium* dinoflagellates), and coral cells (reviewed within [3]). While *Symbiodinium* are critical for providing carbon to the coral [4], bacteria and archaea may also play important roles by enhancing nutrient cycling [5, 6], inducing coral settlement [7], and preventing coral diseases via production of antibiotic compounds [8, 9]. The roles that bacteria and archaea may play in coral health and functioning have encouraged comprehensive investigations into the taxonomic identities and functional genes of microorganisms associated with globally distributed coral species. These studies have described widespread as well as health-related and ecologically important coral-microbial associations [10–12].

Cultivation-independent methods coupled with next-generation sequencing technologies have been increasingly used to examine coral-microbial associations [11–15]. These methods rely on the extraction of nucleic acids (DNA and RNA) from environmental samples and are advantageous because they allow for the study of host-microbe interactions that are difficult to examine using cultivation-dependent methods (reviewed within [3]). The overall utility of these cultivation-independent approaches relies on the comprehensiveness of the extraction of nucleic acids from coral biomass. DNA extraction begins with a series of steps designed to rupture cells using chemical, enzymatic, physical, or mechanical means [16]. Investigators seeking to understand coral-associated microorganisms need to strive for representative lysis of morphologically diverse prokaryotic cells embedded within coral tissue [3, 13, 15, 17] and elution of high-quality nucleic acids.

DNA extraction from coral biomass for investigation of associated bacteria and archaea is particularly subject to technical challenges and potential biases. Coral tissue is rife with polymerase chain reaction (PCR) inhibitors [14, 16, 18, 19], including humic acids and Ca^2+^ ions from the residual coral skeleton. Co-elution of these inhibitors during extraction may decrease PCR efficiency and sensitivity, produce false-negative results [19], delay investigations, and limit comparisons by decreasing sample size. In addition, lysis of microbial cells embedded within the matrix of larger eukaryotic coral cells [3, 13, 15, 17] may be particularly difficult to achieve because of the presence of the mesoglea, a supportive tissue layer comprised of strong collagen fibers that is sandwiched between the epidermal and gastrodermal coral tissue layers [20]. Moreover, lysis affinity for cells of a certain size or structure during sample homogenization could bias interpretation of microbial community composition from sequence-based data [21–23].

Differential lysis of cells during the extraction process may also compound biases associated with PCR amplification. For example, lysis methods with affinities for disrupting coral cells over microbial cells may increase the amount of eukaryotic DNA within the extract, therefore diluting the concentration of microbial relative to eukaryotic DNA. This swamping effect may reduce amplification of microbial DNA during PCR and decrease the overall efficiency of the reaction [24, 25]. In addition, nonspecific amplification of more abundant chloroplast- and mitochondria-derived DNA from the eukaryotic cells by certain primers [25, 26] may distort prokaryotic community structure and lead to exclusion of microbial groups found naturally associated with the coral [27].

Commercial DNA extraction kits offer high-throughput and standardized protocols for streamlined sample processing. As such, using these kits minimizes technical variation and enables researchers to make meaningful comparisons between studies. In particular, kits designed by MO BIO Laboratories, Inc., have been commonly used to extract DNA from coral biomass for downstream analysis [13, 14, 28–37]. However, as described above, not all DNA extracts from coral biomass are amenable to PCR amplification and this may be intensified for particular coral species. Several studies have reported these methodological issues [18, 35, 38], and a few attempts have been made to optimize coral DNA extraction [28, 36, 37, 39, 40]. To date, no large-scale studies have evaluated both the utility of and potential biases associated with different DNA extraction treatments for extraction of DNA from disparate coral species.

In response, we conducted a two-phase experiment in order to (1) propose a comprehensive and efficient method for extraction of microbial DNA from coral tissue and (2) assess if DNA extraction treatment influences microbial community composition. Four commercial DNA extraction kits and protocols supplied by MO BIO Laboratories, Inc. (PowerSoil®, PowerPlant® Pro, PowerBiofilm®, and UltraClean® Tissue & Cells DNA Isolation Kits) were used to extract DNA from seven different coral species during phase I of this study. These kits were selected because they are commonly used to extract DNA from corals [37], and each employs different combinations of chemical, enzymatic, and mechanical disruption to lyse cells. DNA yield and microbial SSU ribosomal RNA (rRNA) gene amplification efficiency were selected as initial screening parameters for phase I extractions because these metrics are inexpensive and quantifiable indicators of DNA extraction success and amplification amenability. The homogenization characteristics of the DNA extraction treatment that yielded the highest average DNA concentrations and amplification efficiencies were further optimized for DNA extraction. These extracts and the second highest performing extracts were then subjected to SSU rRNA gene amplification and sequencing in phase II of this study to investigate potential microbial community bias attributed to the different DNA extraction methods.

## Methods

### Coral collection and processing

Coral fragments were collected by a scuba diver using a hammer and chisel during field sampling trips to Kapangamarangi Atoll, Micronesia (November 2012), the Florida Keys, USA (May 2013), and Magnetic Bay, Australia (November 2013) (Additional file 1: Table S1). Fragments were stored in a cooler containing ice until they were flash frozen in liquid nitrogen. Fragments were obtained from three representative colonies of the following species: *Porites lobata* (collected in Micronesia), *Pocillopora verrucosa* (Micronesia), *Acropora humilis* (Australia), *Orbicella faveolata*, *Montastraea cavernosa*, *Orbicella annularis*, and *Diploria strigosa* (Florida Keys). Fragments were shipped back to Woods Hole Oceanographic Institution and stored at −80 °C until they were processed.

Using an airbrush, an aerosolized jet of autoclaved 1× phosphate-buffered saline (PBS) was directed at freshly thawed coral fragments. This method physically separated the coral mucus and tissue from the skeleton and suspended the cellular material in a slurry. The slurry was homogenized and centrifuged at 4 °C for 20 min (5000 rpm) to form pellets comprised of coral tissue and mucus. The PBS supernatant was removed, and the tissue was evenly divided into smaller sections using an ethanol-sterilized razor. To ensure that differing DNA yields were solely attributed to the lysis efficiency of the extraction treatments, the amount of biomass entering each extraction was standardized for all samples (38.7 ± 9.2 mg). Subsampled biomass fractions were stored in separate tubes and frozen at −80 °C until they were extracted.

### Phase I: DNA extractions

**Fig. 1:**
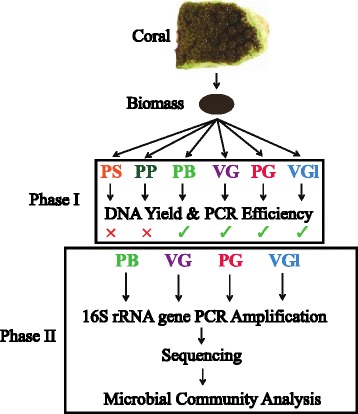
Overview of experimental design. During phase I, DNA extraction treatments were performed on subdivided tissue, with efficiency of SSU gene amplification assessed using gel screening of PCR products. The *green check mark* and *red X* indicate that amplicons from the treatment were and were not chosen for sequencing, respectively. During phase II, well-performing PB, VG, PG, and VGl extracts were amplified and sequenced for microbial community analysis. *PS* PowerSoil, *PP* PowerPlant Pro, *PB* PowerBiofilm, *VG* UC Vortex Garnet, *PG* UC Powerlyzer Glass, *VGl* UC Vortex Glass

DNA was extracted from subsampled coral biomass using the PowerSoil® (cat # 12888), PowerPlant® Pro (cat # 13400), PowerBiofilm® (cat # 24000), and UltraClean® Tissue & Cells (cat # 12334) DNA Isolation kits following the manufacturer’s protocols (MO BIO Laboratories, Inc.) (Fig. 1, Table 1). In this study, the treatments are referred to by their abbreviations: PowerSoil® (PS), PowerPlant® Pro (PP), PowerBiofilm® (PB), and UltraClean® Tissue & Cells (UC). In addition, manipulations to the mechanical lysis conditions for the UC extraction were made, resulting in three permutations: Vortex Garnet (VG), Powerlyzer Glass (PG), and Vortex Glass (VGl) (Table 1). The optional proteinase-K digestion step (15 μl; 20 mg/mL at 60 °C for 30 min) was implemented for all UC permutations. Samples were homogenized for 15 min using a vortex adaptor unless otherwise specified. Genomic DNA concentrations were assessed using the dsDNA High Sensitivity Qubit 2.0 fluorometric assay (Life Technologies). After this study was conducted, MO BIO Laboratories merged with Qiagen and announced plans to rebrand/discontinue some of their products as of January 1, 2017. To ease in this transition, we have provided the original and new names for the kits used in this study: PS is the DNeasy PowerSoil kit, PP is the DNeasy PowerPlant Pro kit, and PB is the DNeasy PowerBiofilm kit. The UC kit has been discontinued.

**Table 1:** Comparison of DNA treatment extraction characteristics

MO BIO extraction characteristics	PS	PP	PB	VG	PG	VGl
Bead diameter (mm)	0.7	2.38	0.1, 0.5, 2.4^a^	0.7	0.1	0.1
Bead type	Garnet	Metal	Glass, Ceramic	Garnet	Glass	Glass
Homogenization method	Vortex	Vortex	Vortex	Vortex	Powerlyzer	Vortex
Homogenization duration	15 min	15 min	15 min	15 min	45 s	15 min
Surfactant	<5%	<5%	X	X	X	X
Protein precipitant	20–40%	10–20%	10–15%	X	X	X
Guanidine thiocyanate	X	<3%	<3%, 60–80%	X	X	X
Inhibitor removal	<10%	<5%	<10%	X	X	X
Proteinase K	X	X	X	1–5%	1–5%	1–5%
RNase	X	25%	X	X	X	X
Phenolic separation solution	X	5–15%	X	X	X	X

*X* indicates that the parameter was not included

*PS* PowerSoil, *PP* PowerPlant Pro, *PB* PowerBiofilm, *VG* UC Vortex Garnet, *PG* UC Powerlyzer Glass, *VGl* UC Vortex Glass

^a^PB kit uses 0.1- and 0.5-mm glass beads and 2.4-mm ceramic beads

DNA template was screened for PCR efficiency using the barcoded primer pair 515F and 806RB [41, 42]. PCR efficiency was determined for each species X treatment pairing as the normalized percentage of successfully amplified amplicons of the correct size (292 bp) out of all the extracts subjected to PCR. To assess PCR efficiency, unaltered DNA template (0.18–47 ng μl^−1^) was amplified in 25 μl reactions containing 1.25 units of GoTaq® Flexi DNA Polymerase (Promega), 5× Colorless GoTaq® Flexi Buffer, 2.5 mM MgCl_2_, 200 μM dNTP mix, and 200 nM of each barcoded primer in a thermocycler (Bio-Rad Laboratories). The following PCR reaction conditions were used: 95 °C for 2 min, followed by 40 cycles of 95 °C for 20 s, 55 °C for 15 s, and 72 °C for 5 min, concluding with an extension step of 72 °C for 10 min. PCR products were visually screened electrophoretically for quality using a 1% agarose and tris-borate-EDTA gel illuminated with ultraviolet light with the Hyperladder 50 bp DNA ladder (5 ng μl^−1^) (Bioline). Positive amplification for each tested extract was denoted by the presence of a 292-bp-sized band.

### Mechanical lysis modifications

The extraction treatment that yielded extracts with the highest PCR efficiency for all coral colonies and species (UC) was selected to further examine if differences in bead type, homogenization method, and homogenization duration resulted in intra-treatment extraction biases (Fig. 1, Table 1). The garnet beads provided with the UC kit were replaced with 0.1-mm glass beads (cat # 13118, MO BIO Laboratories, Inc.) (VGl). For the second modification, a PowerLyzer® 24 bench-top bead-based homogenizer (MO BIO Laboratories, Inc., cat # 13155) was used to homogenize the tissue instead of the vortex adaptor, and garnet beads were replaced with 0.1-mm glass beads (PG). Samples were homogenized with the Powerlyzer for 45 s at 3500 rpm. DNA was not extracted from 3 of the 22 colonies (2 *P. verrucosa* and 1 *A. humilis*) using the VGl treatment because of limited biomass. DNA concentrations were quantified, and PCR efficiency was assessed using the methods outlined above.

### Phase II: library preparation and sequencing

Amplicons obtained from the PB, VG, PG, and VGl extractions were selected for sequencing based on overall DNA yield and PCR efficiency (Table 2). In addition, two positive DNA controls obtained from *Escherichia coli* (Promega) and the Human Microbiome Project mock community DNA (BEI Resources, cat # HM-276D) and a negative control (U.V. sterilized DNA-free water) were amplified, barcoded, and included in the library pool. As an extra assessment of barcode reproducibility, each *O. annularis* extract was assigned two unique barcodes, amplified in separate reactions, and sequenced.

**Table 2:** Summary of DNA extraction yield and PCR efficiency for extractions performed in phase I, with samples selected for phase II bolded

Treatment × species (number of samples)	Average DNA yield (S.D.) (ng μl^−1^)^a^	PCR efficiency^d^
PS × *P. lobata* (3)	4.38 (2.60)^b, c^	1
PP × *P. lobata* (3)	3.73 (1.92)^b, c^	0.67
**PB** × ***P. lobata*** **(3)**	**3.27 (2.86)** ^**b, c**^	**1**
**VGl** × ***P. lobata*** **(3)**	**0.74 (0.36)** ^**c**^	**1**
**PG** × ***P. lobata*** **(3)**	**0.40 (0.06)** ^**c**^	**0.33**
**VG** × ***P. lobata*** **(3)**	**10.31 (5.69)** ^**b**^	**0.67**
PS × *P. verrucosa* (3)	7.35 (10.71)	0.67
PP × *P. verrucosa* (3)	7.96 (7.81)	0
**PB** × ***P. verrucosa*** **(3)**	**27.21 (23.58)**	**0.67**
**VGl** × ***P. verrucosa*** **(1)**	**4.14**	**1**
**PG** × ***P. verrucosa*** **(3)**	**6.06 (3.56)**	**1**
**VG** × ***P. verrucosa*** **(3)**	**12.17 (6.83)**	**1**
PS × *A. humilis* (3)	18.11 (25.20)	0
PP × *A. humilis* (3)	7.75 (11.33)	0.33
**PB** × ***A. humilis*** **(3)**	**38.20 (11.70)**	**1**
**VGl** × ***A. humilis*** ** (2)**	**9.46 (5.43)**	**1**
**PG** × ***A*** **.** ***humilis*** **(3)**	**5.49 (4.56)**	**1**
**VG** × ***A. humilis*** **(3)**	**29.33 (3.31)**	**1**
PS × *O. faveolata* (4)	6.51 (2.95)	0.75
PP × *O. faveolata* (4)	2.24 (1.46)	0
**PB** × ***O. faveolata*** **(4)**	**8.05 (8.08)**	**1**
**VGl** × ***O. faveolata*** **(4)**	**8.24 (7.69)**	**1**
**PG** × ***O. faveolata*** **(4)**	**7.47 (9.79)**	**1**
**VG** × ***O. faveolata*** **(4)**	**12.19 (13.27)**	**1**
PS × *M. cavernosa* (3)	2.73 (2.10)	0.33
PP × *M. cavernosa* (3)	1.31 (0.42)	0
**PB** × ***M. cavernosa*** **(3)**	**1.62 (1.06)**	**0.67**
**VGl** × ***M. cavernosa*** **(3)**	**0.67 (0.36)**	**0.67**
**PG** × ***M. cavernosa*** **(3)**	**1.59 (1.58)**	**1**
**VG** × ***M. cavernosa*** **(3)**	**1.79 (1.73)**	**0.33**
PS × *O. annularis* (3)	7.23 (7.54)	0
PP × *O. annularis* (3)	1.97 (1.18)	0
**PB** × ***O. annularis*** **(3)**	**9.92 (7.31)**	**1**
**VGl** × ***O. annularis*** **(3)**	**1.63 (1.08)**	**1**
**PG** × ***O. annularis*** **(3)**	**1.47 (1.78)**	**1**
**VG** × ***O. annularis*** **(3)**	**11.47 (13.35)**	**1**
PS × *D. strigosa* (3)	2.28 (2.25)	0.33
PP × *D. strigosa* (3)	1.14 (0.29)	0
**PB** × ***D. strigosa*** **(3)**	**0.96 (0.62)**	**0.33**
**VGl** × ***D. strigosa*** **(3)**	**0.24 (0.16)**	**0.33**
**PG** × ***D. strigosa*** **(3)**	**0.33 (0.14)**	**0.33**
**VG** × ***D. strigosa*** **(3)**	**0.98 (0.41)**	**1**

^a^Superscripts (b, c) within a column indicate significantly different DNA concentrations between treatments (one-way FRMANOVA; Holm-Sidak method, *p* < 0.05)

^d^Values represent normalized PCR efficiency

DNA template was amplified with the same V4 primer set using similar PCR reaction conditions to those described above, but with the number of cycles reduced to 35. Amplicons were purified using gel purification (MinElute PCR Purification Kit, Qiagen) so that only PCR products matching the approximate size of the V4 SSU rRNA gene amplicon were included in the final library pool. Samples were prepared for sequencing using the methods previously outlined by Apprill and colleagues [41]. The amplicon pool was shipped to the University of Illinois W. M. Keck Center for Comparative and Functional Genomics and sequenced using 2× 250 bp MiSeq (Illumina) [41, 42].

### Sequence processing

Mothur software [42] (v.1.33.3) was used to combine the de-multiplexed paired reads (8,344,281 contigs) and remove longer sequences (>275 bp) and sequences containing ambiguous base pairs. The expected length of the amplified region with the PCR-specific primers removed was 254 bp. A subset of longer sequences with read lengths exceeding 275 bp were queried using the NCBI BLASTN 2.3.0 program [43, 44] to evaluate the taxonomic affiliation of these sequences. The remaining sequences were classified using the SILVA SSU Ref database [45] (v. 119), and sequences corresponding to Eukaryota, mitochondria, and “unknown” lineages were discarded (2802 sequences). Chloroplast sequences were retained to assess if more chloroplast sequences were associated with a particular DNA extraction treatment. The UCHIME algorithm [46] was used to identify and remove chimeric sequences (13,724 sequences total). Sequences were not subsampled [47, 48].

The sequences were grouped into nodes using the Minimum Entropy Decomposition (MED) algorithm [49]. These MED nodes are analogous to operational taxonomic units (OTUs) and resolve biologically meaningful and distinct groups that can be separated by <1% sequence disparities [49, 50]. Taxonomy was assigned to MED nodes using the classify.seqs command in mothur [42] and the SILVA database (v. 119) [45]. Sequences belonging to “unclassified” MED nodes were re-aligned using the SINA alignment service [51] (v. 1.2.11) and imported into ARB [52] using the SILVA v. 123 database where phylogenetic comparisons were made using neighbor joining algorithms to resolve “unclassified” taxonomy. The mock community and positive control DNA sample yielded the expected communities, replicate barcoded samples produced repeatable results, and the negative control samples did not pass quality control; these samples were then excluded from the analysis.

### Statistical analysis

DNA concentrations were tested for normality using the Shapiro-Wilk test. Concentrations were then subjected to one-way analysis of variance (ANOVA) or Friedman repeated measures analysis of variance (FRMANOVA) on ranks tests, if data failed the Shapiro-Wilk test, to assess if there were significant differences in mean DNA concentrations between coral species or DNA extraction methods. When appropriate, Tukey’s, Holm-Sidak, or Dunn’s method post hoc tests were used to determine significantly different groups. *p* values ≤0.05 were accepted as being statistically significant. The above statistical tests were conducted using SigmaPlot software (v. 13).

Primer (v.7.0.9, Primer- E Ltd.) was used for a majority of the microbial community visualization and alpha diversity analysis. MED richness was calculated using the average number of unique MED nodes detected for each species × treatment grouping. MED species evenness was determined using the averaged Pielou’s evenness index (J’). Bray-Curtis distances were calculated from normalized, square-root transformed sequence data and used to conduct non-metric multidimensional scaling (nMDS) and nested two- and one-way analysis of similarity (ANOSIM) tests. Presence/absence heat maps of MED nodes detected from *O. faveolata*, *O. annularis*, and *A. humilis* associated amplicons were created using the phyloseq [53] R package and a custom script [54] that was modified for this study. These heat maps were generated using distinct MED nodes that comprised 50% of all the reads obtained for each sample and thus represent the most dominant groups found within each colony. Frequency of MED node detection was determined for each treatment (and referred to as the top 50% MED coverage percentage), and the percentage of detection agreement between pairwise treatments within each colony was assessed. One-tailed *t* tests were used to reveal significantly different MED node detection between treatments and were conducted using SigmaPlot.

## Results

### Phase I: DNA yield and PCR efficiency

**Fig. 2:**
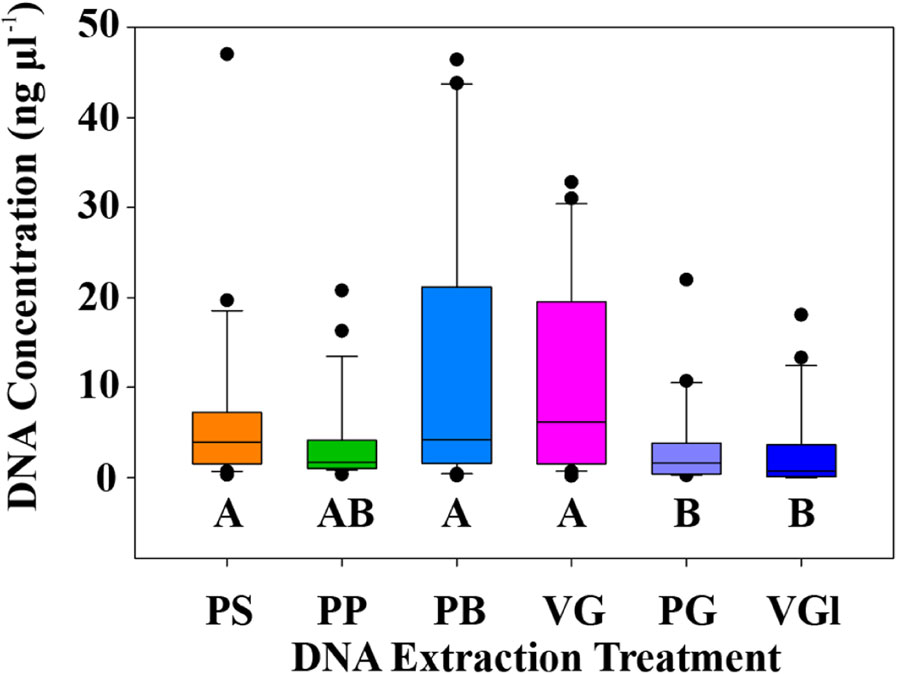
Boxplots of total DNA concentrations grouped by treatment (*n* = 19–22 individual extractions per treatment). *A* and *B letters* differentiate significantly different groups (Tukey’s test *p* < 0.05). Medians are indicated by the *solid black lines*, and the 25 and 75% quartiles are represented by the lower and upper bounds of the box. Outliers are indicated as *black circles* and represent samples falling outside the 10 and 90% quartiles. *PS* PowerSoil, *PP* PowerPlant Pro, *PB* PowerBiofilm, *VG* UC Vortex Garnet, *PG* UC Powerlyzer Glass, *VGl* UC Vortex Glass

DNA concentrations varied among the extraction treatments (PS, PP, PB, VG, PG, and VGl) with PB yielding the highest average concentration of 12.53 ± 15.73 ng μl^−1^ (Fig. 2, Table 2) among all seven coral species. DNA concentrations obtained using the PB and VG treatments had wider distributions than the other treatments, ranging from 0.22 to 46.40 and 0.18 to 32.80 ng μl^−1^, respectively. Overall DNA yields from PG and VGl treatments were significantly lower than yields from the PB, VG, and PS treatments (Fig. 2, FRMANOVA, df = 5, *p* < 0.001; Tukey’s test, *p* < 0.05). Assessment of DNA yields by coral species revealed that PG and VGl *P. lobata* extracts had significantly lower DNA yields than VG extracts (Table 2; one-way FRMANOVA; Holm-Sidak method, *p* < 0.05), but this trend was not observed for the other species.

Gel screening was used to assess the efficiency of SSU rRNA gene amplification; VG and VGl extracts had the highest species coverage and PCR efficiency, defined as the percentage of extracts yielding visible and appropriately sized (~292 bp) bands in the gel (Table 2, Additional file 1: Table S2). Similarly, efficiencies of PB and PG extracts were moderately high (amplifying for 82% of all of the samples) and comparable with the VG and VGl extracts (Additional file 1: Table S2). In contrast, efficiencies of PS and PP extracts were poor with the PS extracts amplifying for 45% of samples and PP extracts only amplifying for 14% of all samples (Additional file 1: Table S2). Nonspecific priming, indicated by the presence of multiple larger or smaller bands, was evident in a majority of the samples regardless of treatment. These nonspecific bands (~200 and ~450 bp) were prominent in 58 and 52% of the PCR products derived from PB and VG extracts, respectively.

During library preparation, 26% of the samples failed to amplify using the designated temperature cycling conditions for the primers and 35 PCR cycles. Some of these samples may have amplified at a higher number of PCR cycles or with dilution of the DNA template, but PCR optimization for every extract extended beyond the goals of this experiment. DNA extracts obtained from *D. strigosa* had the highest PCR failure rate of 75% in stark contrast to extracts from *O. faveolata* and *O. annularis* that had 100% PCR amplification success.

### Phase II: sequencing results

**Fig. 3:**
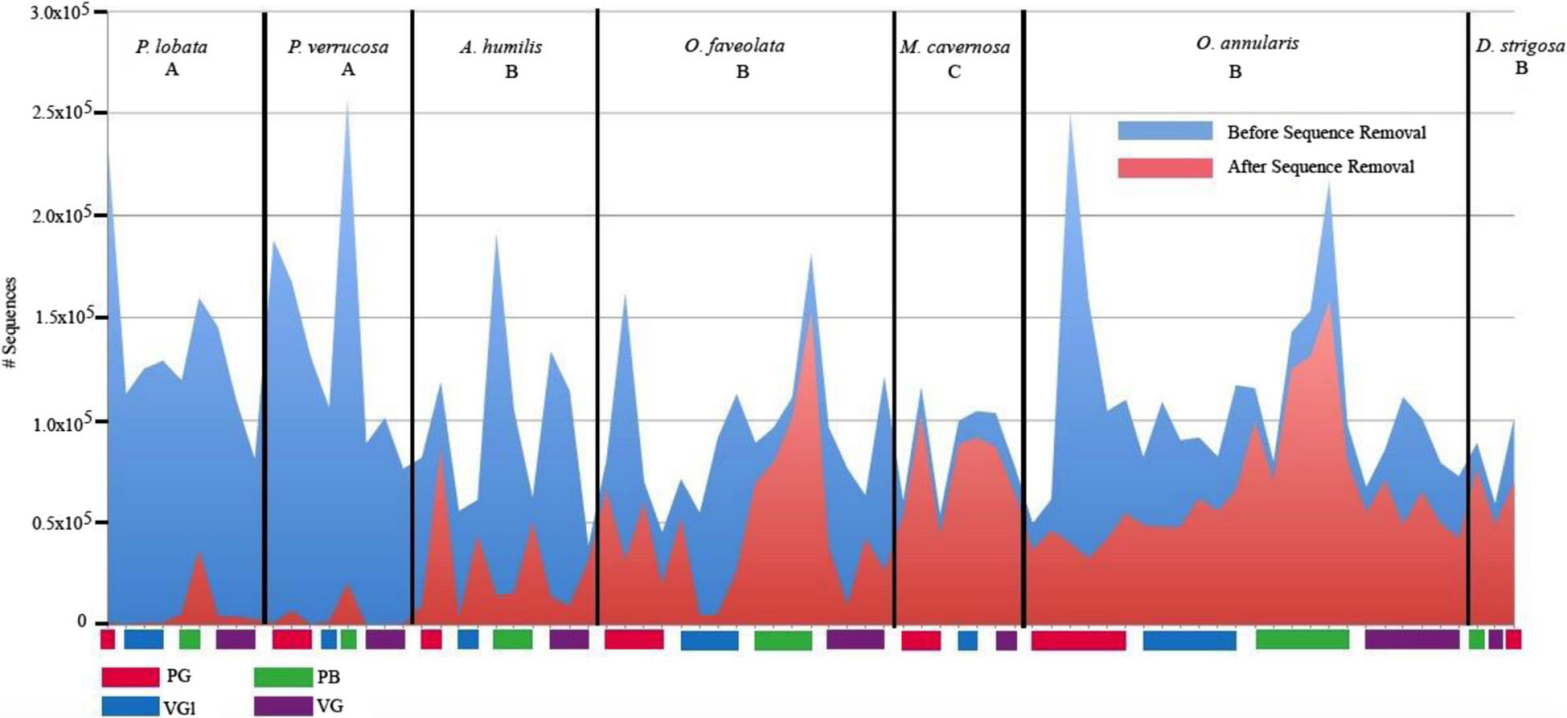
Number of sequences before and after quality filtering and removal of low-quality sequences. Samples are grouped by DNA extraction treatment nested within coral species. *Different letters* (*A*, *B*, and *C*) denote statistically significant differences between species (one-tailed *t* test or Mann-Whitney ranked sums test, *p* < 0.05). *PG* UC Powerlyzer Glass, *VGl* UC Vortex Glass, *PB* PowerBiofilm, *VG* UC Vortex Garnet

Amplicons obtained from the PB, VG, PG, and VGl treatments were prepared for sequencing of SSU rRNA genes in order to thoroughly assess the impact of DNA extraction treatment on microbial community composition. These amplicons were generated from 65 discrete coral colony and extraction treatment combinations representing all seven coral species. Regardless of treatment, there was a statistically significant disparity in the number of quality-filtered microbial sequences obtained from *P. lobata* and *P. verrucosa* corals in comparison to the other species (Fig. 3). A majority of the reads obtained from *P. lobata* and *P. verrucosa* amplicons were too long and therefore were eliminated during preliminary quality-filtering. A subset of these longer reads corresponded to mitochondrial coral DNA sequences (NCBI accession numbers for top identities: JQ911534.1, *e* value = 1e−102; EF597054.1, *e* value = 1e−102; LN864762.1, *e* value = 6e−101). *P. lobata* and *P. verrucosa* amplicons contributed a smaller proportion of sequences to the dataset in comparison to other species because of these disparities. In addition, all *M. cavernosa* PB extracts and *D. strigosa* VGl extracts had very low reads from the outset and were removed from analysis during quality filtering.

**Fig. 4:**
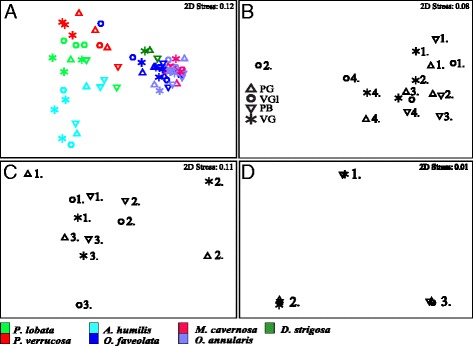
nMDS ordination of SSU rRNA gene sequences recovered from the different DNA extraction treatments and compared using Bray-Curtis distances for **a** all species, **b** *O. faveolata*, **c** *O. annularis*, and **d** *A. humilis*. In **a**, species groupings are designated by *colors*. In **b**–**d**, samples from the same coral colony are designated by number

Microbial community analysis of the SSU rRNA gene sequences demonstrated that, on a large-scale, microbial community composition was significantly influenced by coral species and not DNA extraction treatment (Fig. 4a, two-way nested ANOSIM, seven coral species (B) nested within four extraction treatments (A), for A: *R* = −0.059, *p* = 0.798, for B: *R* = 0.684, *p* = 0.001). Independent analysis of *O. faveolata*, *O. annularis*, and *A. humilis* amplicons (Fig. 4b–d) revealed that microbial community composition was regulated by the coral colony used in the extraction (two-way nested ANOSIM, coral colonies (B) nested within four extraction treatments (A), for A: *R* = 0.013, *p* = 0.267, for B: *R* = 0.9, *p* = 0.001) rather than DNA extraction treatment. Additional testing within each coral species confirmed this observation that DNA extraction method did not significantly influence microbial community composition (one-way ANOSIM, *R* = 0.022, *p* = 0.364, *O. faveolata*; *R* = 0.065, *p* = 0.239, *O. annularis*; *R* = −0.044, *p* = 0.512, *A. humilis*). Non-metric multidimensional scaling analysis further supported this result, demonstrating that the same coral colonies clustered together regardless of the extraction treatment (Fig. 4b–d). In-depth microbial community analysis was not possible for all species and treatments because of PCR inhibition and sequence disparities (Table 3).

**Table 3:** Summary of microbial community analysis conducted during phase II

Treatment × species	*n*	# reads^a^	MED richness^b^	MED evenness (J’)^c^	Total community structure^d^	Detailed analysis	Top 50% MED coverage
PB × *P. lobata*	2	142,167 (14,141)	129 (35)	0.40 (0.13)	VGl, PB: *R* = 0.75 VGl, VG: *R* = 1 VG, PB: *R* = 0.5 *p* = 0.001 for all	No; low reads	–
VGl × *P. lobata*	3	401 (161)	86 (22)	0.70 (0.07)^d^			
PG × *P. lobata*	1	641	100	0.61	–		
VG × *P. lobata*	3	2594 (713)	74 (16)	0.29 (0.08)	Tested above		
PB × *P. verrucosa*	1	13,153	161	0.53	–	No; low reads; low biological replication	–
VGl × *P. verrucosa*	1	603	117	0.78	–		
PG × *P. verrucosa*	3	745 (691)	105 (19)^d^	0.77 (0.16)	No differences		
VG × *P. verrucosa*	3	152 (23)	65 (11)	0.85 (0.04)			
PB × *A. humilis*	3	22,105 (20,156)	131 (30)	0.31 (0.21)	No differences	Yes	0.78
VGl × *A. humilis*	2	21,133 (27,005)	60 (2)^d^	0.36 (0.41)			0.67
PG × *A. humilis*	2	42,241 (53,217)	85 (2)	0.40 (0.45)			0.67
VG × *A. humilis*	3	15,536 (11,314)	59 (5)^d^	0.27 (0.21)			0.7
PB × *O. faveolata*	4	72,587 (25,401)	378 (71)	0.60 (0.16)	No differences	Yes	0.84
VGl × *O. faveolata*	4	16,355 (18,421)	237 (90)	0.56 (0.17)			0.71
PG × *O. faveolata*	4	28,852 (17,895)	392 (87)	0.66 (0.12)			0.77
VG × *O. faveolata*	4	18,630 (9437)	361 (43)	0.61 (0.10)			0.68
PB × *M. cavernosa*	0	n/a	n/a	n/a	n/a	No; not all treatments represented	–
VGl × *M. cavernosa*	2	65,040 (3908)	405 (22)	0.63 (0.12)	No differences		
PG × *M. cavernosa*	3	51,741 (26,956)	366 (40)	0.57 (0.04)			
VG x *M. cavernosa*	2	52,055 (7028)	355 (115)	0.67 (0.14)			
PB × *O. annularis*	3	87,777 (11,390)	485 (60)	0.73 (0.09)	No differences	Yes	0.87
VGl × *O. annularis*	3	32,681 (4358)	357 (45)	0.74 (0.08)			0.77
PG × *O. annularis*	3	24,582 (4974)	313 (130)	0.75 (0.15)			0.67
VG × *O. annularis*	3	34,152 (8088)	382 (123)	0.71 (0.16)			0.77
PB × *D. strigosa*	1	65,392	268	0.27	n/a	No; not all treatments represented; low biological replication	–
VGl x *D. strigosa*	0	n/a	n/a	n/a			
PG × *D. strigosa*	1	60,393	211	0.23			
VG × *D. strigosa*	1	41,726	172	0.18			

All values are presented as mean (standard deviation (S. D.)) when appropriate. Single values with no S.D. represent samples from treatments with no replicates, and these values were not included in statistical significance testing

*n* = number of samples included in microbial community analysis after quality-filtering sequences

^a^Average number of reads obtained for that species × treatment grouping out of the total number of analyzed reads

^b^Species superscripts within a column indicate significantly different MED richness between treatments (*p* < 0.05, one-tailed *t* test or one-way ANOVA with the Holm-Sidak method post hoc test)

^c^MED evenness values (J’) for each treatment with different superscripts indicate significant differences in species evenness (*p* < 0.05, one-way ANOVA with Holm-Sidak method post hoc test)

^d^Differences in community structure were first determined using one-way ANOSIM global tests within each coral species (*p* < 0.05 is significance threshold). If significant differences were found, pairwise tests were conducted between the different treatments. Species × treatment combinations with only one sample were excluded in this analysis

Overall, no statistically significant differences in MED node richness were detected for a majority of the treatments, with the exception of amplicons from *P. verrucosa* (df = 4, one-tailed *t* test, *p* = 0.03) and *A. humilis* (df = 2, one-way ANOVA, *p* = 0.023) (see Table 3). For *A. humilis*, Holm-Sidak method tests revealed higher MED node richness in PB compared to VG (*p* = 0.039) and VGl (*p* = 0.041) amplicons. Generally, the average number of MED nodes was lower for *P. lobata*, *P. verrucosa*, and *A. humilis* amplicons in comparison to the other species. Furthermore, amplicons generated from the PB treatment were more likely to have the highest MED richness out of the four treatments, with this being the case for five of the seven species (Table 3). MED species evenness (J’) tended to be higher in all treatments except the PB treatment (Table 3). Overall, *D. strigosa* had the lowest evenness whereas *O. annularis* and *P. verrucosa* had the highest evenness.

**Fig. 5:**
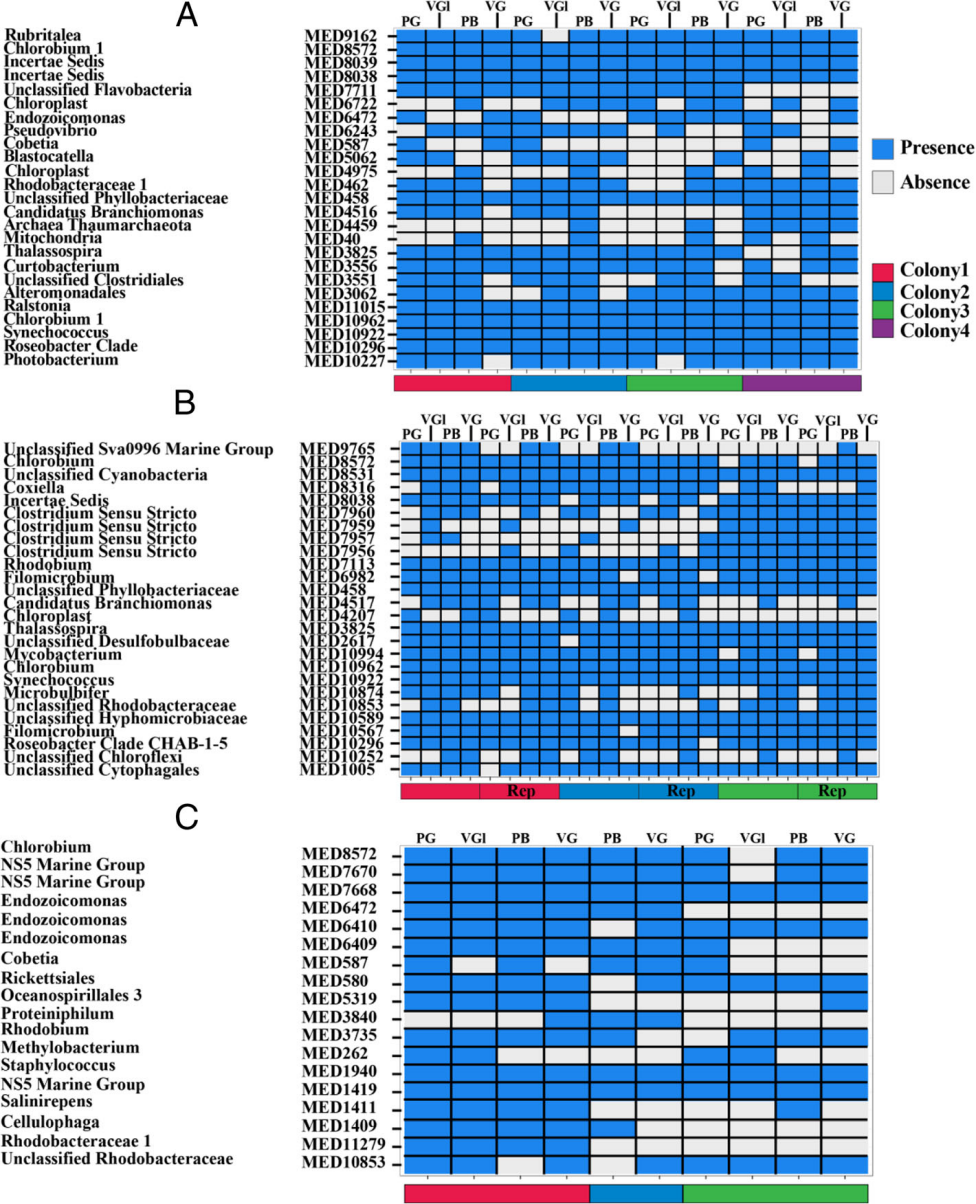
Heatmaps displaying the presence or absence of dominant MED nodes that ranked within the top 50% of the dataset for each species for **a** *O. faveolata*, **b** *O. annularis*, and **c** *A. humilis.* ‘Rep’ designates technical replicates of identical *O. annularis* DNA extracts tagged with different barcodes during PCR. The *colors* designate different colonies of that species

To tease apart the pattern between MED richness and DNA extraction method on a finer scale, pairwise investigations of differences between treatments were completed using presence/absence analysis of MED nodes for *O. faveolata*, *O. annularis*, and *A. humilis* (Fig. 5). Overall, there was fairly high MED node detection agreement between the sequences generated from different treatments extracted from the same coral colony (average agreement ranged from 72–85%). Closer inspection revealed that *O. faveolata* PB sequences had significantly higher MED presence/absence coverage of dominant microbial groups compared to VGl (one-tailed, paired *t* test, *p* = 0.013) and VG (*p* = 0.007) sequences. Including technical replicates, PB sequences from *O. annularis* had significantly higher MED coverage compared to sequences from the PG (*p* = 2.0 × 10^−4^) and VGl (*p* = 0.019) treatments. A significant difference in MED coverage was revealed between PG and VGl amplicons (*p* = 0.004) for *O. annularis*, but this trend was not observed in *O. faveolata* or *A. humilis*. Sequences generated from *O. faveolata* PB extracts contained more “*Candidatus* Branchiomonas” (MED node 4516) and *Thaumarchaeota* (MED node 4459) reads in comparison to other treatments from this species. Similarly, *Ca.* Branchiomonas (MED node 4517) was identified in more PB treatment sequences from *O. annularis* compared to the other treatments. MED node presence/absence agreement in *O. annularis* technical replicates was very high with only a few occurrences of disagreement between three technical replicates (9 out of 182 possible disagreements). Statistically significant differences in MED coverage of the dominant groups between treatments were not detected in sequences obtained from *A. humilis* amplicons, but sequences from the PB treatment had the highest coverage of dominant MED nodes (78%) out of all the treatments for this species.

## Discussion

In this study, the PB and all variations of the UC (PG, VGl, VG) treatments were found to be technically suitable and reliable for extraction of microbial DNA from most colonies of *P. lobata*, *P. verrucosa*, *A. humilis*, *O. faveolata*, *O. annularis*, and *D. strigosa*. PCR inhibition during library preparation and significant removal of sequences during quality-filtering prevented highly resolved comparisons for *P. lobata*, *P. verrucosa*, *M. cavernosa*, and *D. strigosa*, demonstrating the importance of including many biological replicates for each species in sequencing-based investigations. Broadly, extraction treatment did not significantly bias microbial community composition, but finer scale investigations for *O. faveolata*, *O. annularis*, and *A. humilis* revealed minor differences in MED coverage and group sensitivity between the UC and PB derived communities.

Generally, all treatments yielded DNA concentrations that fell within the range of previously reported DNA concentrations for corals [36, 37]. While the VG and PB treatments yielded similar DNA concentrations, the PG and VGl treatments yielded extracts with lower DNA concentrations, an observation likely attributed to differences in the duration of mechanical lysis and bead size. In this study, significantly higher DNA yields were obtained from treatments that homogenized samples for 15 min (VG, PB) on a vortex adaptor in comparison to 45 s (PG) using a powerlyzer and this result aligns with the reported observation that DNA concentration increases with bead-beating duration [55]. Furthermore, larger beads are more likely to lyse eukaryotic coral cells and release more DNA (~420 Mbp coral genome^−1^ [56]) whereas smaller beads are probably targeting the smaller microbial cells containing less DNA (~0.9–9.7 Mbp microbial genome^−1^ [57]). In this study, lower concentrations of DNA may have been obtained because the beads were either too large in diameter to effectively disrupt coral and microbial cells (PP) or so small that they could not sufficiently rupture eukaryotic cells (PG, VGl). Lastly, the PB and UC treatments had less sample transfer steps (2) than the PS (4) and PP (3) kits. Minimizing steps during extraction likely helps maintain nucleic acid integrity and may also limit introduction of contaminants, reduce waste, and decrease extraction duration.

The UC (VG, PG, and VGl) and PB treatments yielded extracts that had similar PCR efficiencies. This may be because the UC and PB treatments physically lysed cells using high heat exposure [58]. Using an additional method to achieve cellular lysis may have increased the chance of disrupting cells from a wider variety of microorganisms and the overall concentration of prokaryotic DNA relative to eukaryotic DNA within the extraction. More importantly, the 100% amplification success of *O. annularis* UC and PB extracts in this study contrasts with the poor amplification (20–60%) reported for this species in a recent comparative DNA extraction optimization study using the PS and PP DNA treatments [37] and marks a promising advance in defining a suitable extraction method for this species.

PCR inhibition associated with particular coral species (*D. strigosa*) or colonies (*M. cavernosa*) may have arisen due to differences in PCR inhibitor carryover during initial sample processing. For example, we found that the calcium carbonate skeleton of *D. strigosa* colonies fractured more during sample processing in comparison with other species. This likely resulted in co-elution of calcium (Ca^2+^) ions with DNA during the final step of the extraction. Because Ca^2+^ ions compete with magnesium (Mg^2+^) ions as cofactors for DNA polymerase, higher concentrations of Ca^2+^ in *D. strigosa* extracts could have resulted in greater inhibition of DNA polymerase [59, 60]. Baker & Kellogg [37] also offered this hypothesis to explain differential PCR amplification between coral species and emphasized the importance of using multiple coral species for optimization experiments. For future experiments, it may be appropriate to increase the Mg^2+^ concentration used during PCR to overcome this inhibition [59, 60].

Unfortunately, neither DNA concentration nor PCR efficiency alone serve as definite indicators of sequence data quality, a concept not demonstrated in past coral DNA optimization studies [36, 37], but supported by previous coral microbiota sequencing studies [12, 61] and the disparities between DNA concentration, PCR efficiency, and *P. lobata* and *P. verrucosa* sequence quality reported in this study. This observation can possibly be explained by the idea that extracts from *P. lobata* and *P. verrucosa* may be prone to more eukaryotic DNA swamping [37]. Recent efforts for circumventing DNA swamping and nonspecific amplification involve selectively enriching genomic extracts for prokaryotic DNA [24] or designing new PCR primers [62]. Alternatively, as the cost of sequencing declines, deep sequencing of shot-gun metagenomic DNA has increasingly been used to circumvent the issues associated with amplicon-based methods [63]. While this approach may work well for some study systems [64–66], it proves difficult to use for studying complex communities within the coral holobiont; the abundance of coral and *Symbiodinium* genomic material requires deep sequencing and even size-fractionation may not effectively enrich the target communities of interest [11, 67].

Microbial community analysis revealed that most of the variation in microbial community composition corresponded with coral species or colony rather than DNA extraction method. This agrees with the results of a human microbiome study that attributed most of the variation in microbial community composition to different human subjects rather than DNA extraction method [22]. As a whole, this result suggests that the chosen DNA extraction method (either the PowerBiofilm® or the different permutations of the UltraClean® Tissue and Cells DNA Isolation kit) should not impart significant biases on microbial community composition if the aim of the study is to elucidate large differences in microbial community composition that correspond with changes in coral health, coral species, or other factors. Because many investigations are interested in making these larger comparisons, we recommend that both the PowerBiofilm® or the different permutations of the UltraClean® Tissue and Cells DNA Isolation kit are suitable for broad investigations of coral microbial dynamics.

This recommendation is verified by the finding that the dominant taxonomic classes of bacteria and archaea recovered in this dataset support the results of other coral microbiota taxonomic surveys. A recent review from Bourne and colleagues [68] identified Gammaproteobacteria, Alphaproteobacteria, Actinobacteria, Bacteroidetes (esp. Flavobacteria), and Cyanobacteria as common coral-associated bacteria, and all these groups were detected in this study. At a finer scale, we detected bacterial genera that have been previously identified as coral associates. For example, in this study, MED nodes identified as *Endozoicomonas* (class Gammaproteobacteria) were present in all, but two of the samples (varying relative abundances of 0.002–80.02%). *Endozoicomonas* bacteria are recognized as potentially important tissue and mucus associates of corals [50, 68, 69], and their genomes suggest functional adaptations for residing with a host [70, 71]. *Ralstonia* spp. have also been detected in coral microbiota surveys of many different species [13, 28, 69] and observed within coral-host cells in close proximity to symbiotic dinoflagellates [13]. The functional role of *Ralstonia* spp. in corals has not been confirmed, but genetic evidence also suggests that they are well-suited for the symbiotic lifestyle [13]. We detected four distinct MED nodes associated with the *Ralstonia* genus in 74/77 of our samples, with the highest average relative abundances found in *O. faveolata* (2.5 +/− 9.7%) and *Diploria strigosa* (17 +/− 30.1%) corals. This result demonstrates that the DNA extraction methods used in this study may have the capacity to lyse cells located within host-coral cells, thus confirming the use of these DNA extraction methods for studying complex, host-associated microbiomes.

However, if the goal of the investigation is to detect specific or cryptic/rare microorganisms [13], care may need to be taken when choosing the DNA extraction method. This recommendation is supported by the minor, but important distinctions in microbial richness and coverage of top microbial groups between treatments that were detected during pairwise comparisons of the presence/absence of discrete MED nodes by coral colony. For example, nMDS and ANOSIM did not discern the higher MED coverage associated with PB extracts, but this trend was uncovered during presence/absence evaluation. Higher MED coverage, including the increased likelihood of detecting MED nodes not detected in sequences from other extractions (e.g., *Ca.* Branchiomonas, and *Thaumarchaeota*), may stem from using a mixture of bead sizes and types during PB DNA extraction. Crowder and colleagues [72] used a mixture of 0.1- and 2.0-mm beads to extract DNA from ticks and reported that the 2.0-mm beads disrupted the tick exoskeleton, while the 0.1-mm beads disrupted soft tissue and microbial cells. The PB kit also uses a mixture of 0.1- and 0.5-mm glass beads and larger 2.4-mm ceramic beads to mechanically rupture cells. This bead combination may have facilitated lysis of more recalcitrant coral tissue with the ceramic beads (2.4 mm) and lysis of soft coral tissue (0.5 mm) and microbial cells (0.1 mm) with the glass beads. Using bead mixtures during DNA extraction may be particularly important for studies investigating intracellular symbionts or rare microorganisms. Altogether, careful thought about the scope and expected outcomes of the planned research is needed because this may impact which DNA isolation treatment should be used.

## Conclusions

This study demonstrates that the PowerBiofilm® and UltraClean® Tissue and Cells (and permutations) DNA Isolation kits are appropriate to use for extraction and amplification of microbial DNA from most colonies of *P. lobata*, *P. verrucosa*, *A. humilis*, *O. faveolata*, *O. annularis*, and *D. strigosa* corals. Subsequent microbial community analysis revealed that at a large-scale, overall microbial community structure was significantly determined by coral species rather than DNA extraction treatment, a result that validates the use of either the PowerBiofilm® or UltraClean® Tissue and Cells (and permutations) DNA Isolation kits for broad coral microbiota comparisons of globally distributed coral species. On a finer scale, subtle, but potentially important differences in microbial community richness and coverage of top microbial groups were detected, trends that may stem from using different bead mixtures during mechanical lysis of the coral tissue. Based upon these results, we suggest that the PowerBiofilm® DNA extraction kit is the most reliable and comprehensive kit to use for small scale cultivation-independent characterization of coral microbiota.

As reliance on sequence data for scientific inquiry grows, acknowledgement of biases introduced to samples via methods is important not only for investigation of error and replication, but also for the detection of ecologically meaningful patterns. Methods vigilance, obtained by conducting dedicated method optimization studies, is a cornerstone for cultivation-independent investigations of microbe-host associations. Understanding the influence of technical bias aids our detection of biologically relevant patterns from sequence data and deepens our understanding of the coral microbiome as well as other complex host environments.

## Additional file

Additional file 1: Table S1. Provides information about the species designation, collection site, collection depth, and colony names for the coral biomass samples used in this study. Table S2. Provides more detailed information about the PCR efficiency of each sample. (DOCX 125 kb)

## Abbreviations

PB: PowerBiofilm®
PG: UltraClean® Tissue & Cells, PowerLyzer® Homogenizer, Glass Beads
PP: PowerPlant® Pro
PS: PowerSoil®
VG: UltraClean® Tissue & Cells, Vortex Adaptor, Garnet Beads
VGl: UltraClean® Tissue & Cells, Vortex Adaptor, Glass Beads
