# Supplementary material for: Optimization of DNA extraction for advancing coral microbiota investigations
Source: Microbiome. 2017 Feb 8;5:18. doi: 10.1186/s40168-017-0229-y (PMC5299696; doi:10.1186/s40168-017-0229-y)
Supplement: Additional file 1: Table S1. — Provides information about the species designation, collection site, collection depth, and colony names for the coral biomass samples used in this study. Table S2. Provides more detailed information about the PCR efficiency of each sample. (DOCX 125 kb) [file 40168_2017_229_MOESM1_ESM.docx]

Table 1. Coral species, collection site location, collection depth, and colony name for colonies.

| Species | Collection Site | Latitude | Longitude | Depth (ft) | Colony name |
| --- | --- | --- | --- | --- | --- |
| *Porites lobata* | Shallow barrier reef/lagoon, Kapangamarangi Atoll, Micronesia | 1.02695 N | 154.774643 W | 1 | Plob1 |
| *Porites lobata* | Shallow barrier reef/lagoon, Kapangamarangi Atoll, Micronesia | 1.02695 N | 154.774644 W | 1 | Plob2 |
| *Porites lobata* | Shallow barrier reef/lagoon, Kapangamarangi Atoll, Micronesia | 1.02695 N | 154.774645 W | 1 | Plob3 |
| *Pocillopora verrucosa* | Patch reef/lagoon, Kapangamarangi Atoll, Micronesia | 1.0365 N | 154.765717 W | 33 | Pverr1 |
| *Pocillopora verrucosa* | Patch reef/lagoon, Kapangamarangi Atoll, Micronesia | 1.0365 N | 154.765719 W | 33 | Pverr2 |
| *Pocillopora verrucosa* | Patch reef/lagoon, Kapangamarangi Atoll, Micronesia | 1.02695 N | 154.774645 W | 33 | Pverr3 |
| *Acropora humilis* | Magnetic Bay, Australia | 15.0883 S | 146.8520 E | 5 | Ahum1 |
| *Acropora humilis* | Magnetic Bay, Australia | 15.0883 S | 146.8520 E | 5 | Ahum2 |
| *Acropora humilis* | Magnetic Bay, Australia | 15.0883 S | 146.8520 E | 5 | Ahum3 |
| *Diploria strigosa* | Midpatch reef, Fl Keys, USA | 24.59306 N | 81.39111 W | 18 | Dstrig1 |
| *Diploria strigosa* | Openwater patch reef, Fl Keys, USA | 24.557886 N | 81.436053 W | 20 | Dstrig2 |
| *Diploria strigosa* | Midpatch reef, Fl Keys, USA | 24.562519 N | 81.500666 W | 18 | Dstrig3 |
| *Orbicella faveolata* | Nearshore reef, Fl Keys, USA | 24.59306 N | 81.39111 W | 9 | Ofav1 |
| *Orbicella faveolata* | Reef flat, Fl Keys, USA | 24.606054 N | 81.429342 W | 19 | Ofav2 |
| *Orbicella faveolata* | Midpatch reef, Fl Keys, USA | 24.59306 N | 81.39111 W | 18 | Ofav3 |
| *Orbicella faveolata* | Reef flat, Fl Keys, USA | 24.562519 N | 81.500666 W | 17 | Ofav4 |
| *Montastraea cavernosa* | Reef flat, Fl Keys, USA | 24.59306 N | 81.39111 W | 18 | Mcav1 |
| *Montastraea cavernosa* | Openwater patch reef, Fl Keys, USA | 24.557886 N | 81.436053 W | 21 | Mcav2 |
| *Montastraea cavernosa* | Midpatch reef, Fl Keys, USA | 24.562519 N | 81.500666 W | 17 | Mcav3 |
| *Montastraea annularis* | Openwater patch reef, Fl Keys, USA | 24.557886 N | 81.436053 W | 22 | Mann1 |
| *Montastraea annularis* | Midpatch reef, Fl Keys, USA | 24.562519 N | 81.500666 W | 18 | Mann2 |
| *Montastraea annularis* | Openwater patch reef, Fl Keys, USA | 24.557886 N | 81.436053 W | 23 | Mann3 |

Table 2: PCR efficiency displayed for each colony and extraction method.

| Treatment | PS | PP | PB | VG | PG | VGl |
| --- | --- | --- | --- | --- | --- | --- |
| Plob1 | + | + | + | + | + | + |
| Plob2 | + | - | + | - | - | + |
| Plob3 | + | + | + | + | - | + |
| Pverr1 | + | - | + | + | + | X |
| Pverr2 | + | - | - | + | + | + |
| Pverr3 | - | - | + | + | + | X |
| Ahum1 | - | - | + | + | + | + |
| Ahum2 | - | - | + | + | + | X |
| Ahum3 | - | + | + | + | + | + |
| Ofav1 | + | - | + | + | + | + |
| Ofav2 | + | - | + | + | + | + |
| Ofav3 | - | - | + | + | + | + |
| Ofav4 | + | - | + | + | + | + |
| Mcav1 | + | - | + | + | + | - |
| Mcav2 | - | - | - | - | + | + |
| Mcav3 | - | - | + | - | + | + |
| Mann1 | - | - | + | + | + | + |
| Mann2 | - | - | + | + | + | + |
| Mann3 | - | - | + | + | + | + |
| Dstrig1 | - | - | - | + | - | - |
| Dstrig2 | - | - | - | + | - | - |
| Dstrig3 | + | - | + | + | + | + |
| PCR Efficiency◆ | 45% | 14% | 82% | 86% | 82% | 84% |

◆PCR efficiency was calculated as the percentage of successfully amplified bands of the correct size (292 bp, including primers) out of the total number of samples that were subjected to PCR for each extraction treatment.

‘+’= band present, ‘-‘=band absent, ‘X’= no sample.
